# Estimating New Zealand’s harvested wood products carbon stocks and stock changes

**DOI:** 10.1186/s13021-020-00144-5

**Published:** 2020-05-21

**Authors:** Stephen J. Wakelin, Nigel Searles, Daniel Lawrence, Thomas S. H. Paul

**Affiliations:** 1 Scion-New Zealand Forest Research Institute Limited, Rotorua, New Zealand; 2 New Zealand Ministry for the Environment, Wellington, New Zealand

**Keywords:** Greenhouse gas emissions, Harvested wood products (HWPs), Carbon stocks, Stock changes climate change mitigation, IPCC models

## Abstract

**Background:**

Reducing net greenhouse gas emissions through conserving existing forest carbon stocks and encouraging additional uptake of carbon in existing and new forests have become important climate change mitigation tools. The contribution of harvested wood products (HWPs) to increasing carbon uptake has been recognised and approaches to quantifying this pool developed. In New Zealand, harvesting has more than doubled since 1990 while log exports have increased by a factor of 11 due to past afforestation and comparatively little expansion in domestic processing. This paper documents New Zealand’s application of the IPCC approaches for reporting contributions of the HWP pool to net emissions, in order to meet international greenhouse gas inventory reporting requirements. We examine the implications of the different approaches and assumptions used in calculating the HWP contribution and highlight model limitations.

**Results:**

Choice of system boundary has a large impact for a country with a small domestic market and significant HWP exports. Under the Production approach used for New Zealand’s greenhouse gas inventory reporting, stock changes in planted forests and in HWPs both rank highly as key categories. The contribution from HWPs is even greater under the Atmospheric Flow approach, because emissions from exported HWPs are not included. Conversely the Stock Change approach minimises the contribution of HWPs because the domestic market is small. The use of country-specific data to backfill the time series from 1900 to 1960 has little impact but using country-specific parameters in place of IPCC defaults results in a smaller HWP sink for New Zealand. This is because of the dominance of plantation forestry based on a softwood mainly used in relatively short-lived products.

**Conclusions:**

The NZ HWP Model currently meets international inventory reporting requirements. Further disaggregation of the semi-finished HWP end uses both within New Zealand and in export markets is required to improve accuracy. Product end-uses and lifespans need to be continually assessed to capture changes. More extensive analyses that include the benefits of avoided emissions through product substitution and life cycle emissions from the forestry sector are required to fully assess the contribution of forests and forest products to climate change mitigation and a low emissions future.

## Background

Reducing greenhouse gas emissions through conserving forest stocks of carbon and encouraging additional uptake of carbon in existing and new forests have become important tools in climate change mitigation [1]. Forests can play three important roles in the carbon cycle. Firstly, they act as sinks, sources and reservoirs of carbon, interacting with the atmosphere through growth, mortality, and heterotrophic processes. Secondly, they provide harvested wood products (HWPs) that store carbon over the product’s life cycle before returning it to the atmosphere through the processes of decay and combustion. Thirdly, wood and biomass products from forests can be used to substitute fossil fuels—either directly through their use as solid or liquid biofuels or indirectly through substituting for products that produce more greenhouse gas emissions in their production [2].

Under the United Nations Framework Convention on Climate Change (UNFCCC), Annex I Parties are required to submit annual inventories of greenhouse gas emissions and removals from 1990. The Intergovernmental Panel on Climate Change (IPCC) has developed and revised guidance on the estimation of these emissions and removals [3, 4]. The default assumption was initially that wood products removed at the time of harvest should be recorded as an immediate emission of CO_2_, with additions to the HWP pool assumed to be balanced by losses [5]. The 2006 IPCC Guidelines describe two broad methods for estimating emissions and removals in the pools of forests and forest products—the inventory (stock change) method and the flux method. The inventory method estimates the stock of carbon in wood products (e.g. by determining the number of buildings by type and the amount of wood use in each type), and gains or losses from the HWP pool (from the change in stocks over time). The flux method estimates emissions directly by using assumed or identified life spans for products. Four alternative HWP accounting approaches (Stock Change, Production, Atmospheric Flow and Simple Decay) were described that differ in when and where changes in the HWP pool are ascribed. No judgment was made as to which approach is preferred. Instead, the approach taken by the IPCC was to describe how variables related to HWP stocks can be calculated and then combined under each of the alternative accounting approaches. The global estimate will be identical if all countries use the same approach—if not there will be double-counting and/or non-counting of HWPs [4, 6]. For an individual country the approaches lead to very different estimates of the HWP contribution to national net removals, depending on whether the country is a net importer or exporter of HWPs [7].

The Kyoto Protocol (KP) to the UNFCCC strengthened developed country commitments to achieving net emission targets. HWP pool changes were not allowed to contribute towards targets for the first KP commitment period but a restricted version of the Production Approach was adopted for all Parties for the second commitment period [8].

**Fig. 1:**
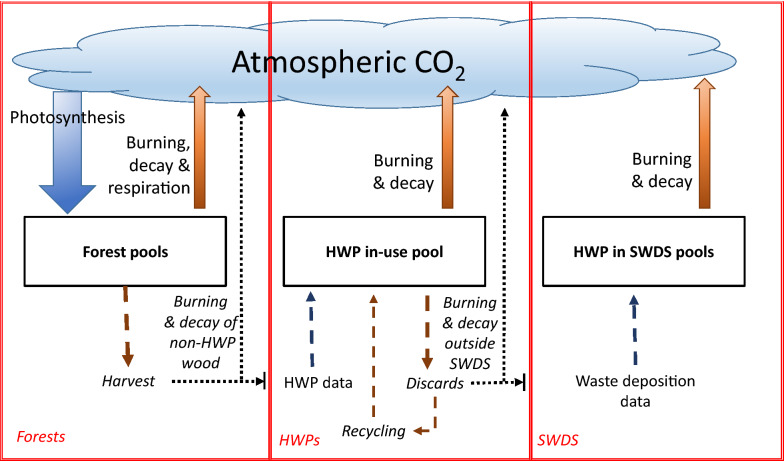
IPCC GHG inventory stocks and flows. Solid arrows are actual emissions and sinks (fluxes to and from the atmosphere); dashed arrows are stock changes effectively treated as emissions or sinks by the three models; dotted arrows are emissions implied through their exclusion from the reported carbon stocks

The default (Tier 1) IPCC methodology does not require tracking of the full life cycle of carbon from trees through products to disposal and ultimate return to the atmosphere. Figure 1 shows how the IPCC Tier1 methods covering carbon in forests, HWPs in use, and HWPs in landfills (solid wood disposal sites, SWDS) are conceptually—but not explicitly—linked. The only true sink process is photosynthesis—individual pools within the forest (i.e. above-ground biomass, below-ground biomass, dead wood, litter and soil organic carbon) can increase due to transfers from other pools (e.g. from above-ground biomass to deadwood), but overall gains are only due to carbon uptake by biomass through photosynthesis, the single most significant process to extract CO_2_ from the atmosphere. Similarly, carbon is returned to the atmosphere from the forest pools only through burning, decay and autotrophic and heterotrophic respiration. Tree harvesting is a transfer of carbon to pools that may be within the forest (i.e. harvest residues) or outside (extracted logs). The IPCC stock change-based approaches treat the removal of logs from the forest as an instant emission rather than a transfer, balanced to some extent by treating the inflow of sawn timber, panels and paper into the HWP pool as a sink. Wood that is not incorporated into these qualifying HWPs (including wood used for bioenergy) is implicitly treated as an emission, captured by the annual forest stock change due to harvesting less the inflow to the HWP pool. Tier 1 UNFCCC reporting recognises solid wood and paper and paperboard as the two semi-finished products to be reported, while the Kyoto Protocol separates solid wood into sawn wood and wood-based panels.

Implementation of the Tier 1 methods provided by the IPCC does not track carbon extracted by harvesting through to the HWP pool or further (e.g. to discarded HWPs being deposited into landfills). Instead different data sets are used as inputs to the HWP pool (FAO production and trade data) and to the SWDS model (wood deposition in landfills data). In their review of HWP models, Jasinevičius et al. described this as applying “bookkeeping principles” [9]. An alternative would be to estimate just the true emission processes that return harvested carbon to the atmosphere (see Fig. 1), on the assumption that the remainder of the harvest remains incorporated within HWPs. Rather than attempting to model the life spans of products in use, this would require estimates of:

- carbon in harvested logs taken off site;
- carbon released by wood burned for energy;
- carbon released from wood not burned for energy or captured in HWPs (e.g. animal bedding);
- carbon released from decay or combustion of HWPs in use (e.g. building fires);
- carbon released from wood deposited into solid waste disposal sites;
- carbon released from HWPs discarded outside solid waste disposal sites.

While some of these flows are already reported in the GHG inventory, others may be no more easily estimated than HWP discard rates. Tier 1 and 2 methods attempt to simplify data requirements.

In New Zealand almost all (99.8% in 2018) of the 30 million m^3^ annual harvest is from managed planted forests, 90% of which are even-aged stands of a single species—*Pinus radiata* D. Don [10]. Carbon stocks in New Zealand’s planted forests and the HWPs derived from them were first estimated by Maclaren and Wakelin [11]. The HWP pool was modelled by assuming that the same mix of products would continue to be produced from the annual harvest, divided into long, medium and short-lived products with assumed lifespans of 80, 50 and 1 year respectively. The extended life span of HWPs in landfills was ignored, and the HWP stock was initialised in 1990 based on an estimation of the quantity of wood in the housing stock and the ratio of wood use in housing to wood used in other applications. Thus, the HWP pool was estimated by a mix of bookkeeping flux methods and inventory stock methods.

Subsequently the IPCC HWP spreadsheet model [3] was adapted by New Zealand for reporting under the UNFCCC and the contribution of HWPs to net emissions was first reported as part of New Zealand’s 1990–2013 Greenhouse Gas Inventory [12].

New Zealand’s greenhouse gas profile is unusual for a developed country, with a high contribution from renewables to electricity generation, half of gross emissions coming from the Agriculture sector and significant offsetting of emissions by fast-growing planted forests on relatively short rotations. The LULUCF sector offset about one-third of gross emissions from 1990 to 2017 and the annual harvest has more than doubled since 1990 as a result of historic afforestation [13]. Accordingly, stock changes in planted forests and in the HWP pool (estimated with the Production approach) ranked highly as key categories in terms of both the level and trend in New Zealand’s 1990 to 2017 GHG inventory [13] (Table 1).

**Table 1:** 1990–2017 Key category analysis—forests and HWPs

Category	GHG	Level		Trend	
		Rank	% contribution	Rank	% contribution
Land converted to forest land	CO_2_	1st	14.3	5th	10.2
Forest land remaining forest land	CO_2_	6th	4.9	3rd	11.1
Land use, land-use change and forestry—harvested wood products	CO_2_	5th	5.7	6th	7.0

While the HWP pool is an important component of New Zealand’s GHG inventory it is also unusual in its composition as over half of the annual harvest is exported as raw materials (logs, wood chips or pulp) and a high proportion of HWPs produced in New Zealand are also exported (approximately half for each of sawn timber and panels and three-quarters of paper). Imported HWPs make up about one-third of domestic apparent consumption (roundwood equivalent), of which over 80% is pulp and paper [29]. This means that the choice of system boundary for accounting (i.e. whether exported or imported HWPs are included) has a large impact on reported net emissions. Since New Zealand is expected to continue to rely in part on forest offsets to meet current and future net emission targets [14], HWPs will remain an important part of New Zealand’s GHG inventory.

This paper presents New Zealand’s application of the IPCC approaches for reporting contributions of the HWP pool to net emissions, in order to meet international greenhouse gas inventory reporting requirements. We examine the implications of the different approaches and assumptions used in calculating the HWP contribution and highlight limitations of the model.

## Methods

This paper describes the HWP model developed and applied for reporting the contribution of the HWP pool to New Zealand’s net emissions under the UNFCCC and KP. Four approaches were implemented using New Zealand data where available, including the three approaches for UNFCCC reporting (Stock-change, Atmospheric flow and Production) and the restricted version of the production approach in accordance with guidelines for KP reporting [3, 4, 8]. A fifth approach—Simple Decay—was also implemented but is omitted here for clarity. This is because the 2006 IPCC Guidelines recommended applying the ‘simple decay’ function to semi-finished HWPs, making it equivalent to the Production approach. The alternative is to apply a decay function directly to the harvest, reflecting both the discard of HWPs in use and the lifetime of harvested material that is never incorporated into HWPs. This is then not directly comparable to the other three approaches which all apply discard rates to semi-finished products but differ in system boundary. The impact of several assumptions regarding activity data and emission factors is assessed through scenario analysis and an assessment of overall model uncertainty is provided.

### NZ HWP model structure

The IPCC HWP Tier 1 model estimates stock changes since 1900 in the “in-use” pools of two product categories (solid wood and paper), calculated using first order decay functions. The main inputs are Food and Agriculture Organisation (FAO) data on HWP production, exports and imports since 1961, factors for converting product quantities to carbon and product half-lives. The model estimates the variables required for reporting under the alternative accounting approaches and assumes that export logs will be converted into the same mix of products as with domestic processing with the same half-lives. This model was adapted in 2008 by New Zealand for reporting under the UNFCCC and for policy analysis. The main changes to the original model made initially were to extend projections to 2030, to include the original Simple Decay approach (applied to harvest removals directly) and to incorporate the country-specific parameters that were available at the time, such as mean wood density (i.e. Tier 2).

A separate spreadsheet model was developed to report HWPs under the KP according to IPCC guidance [8]. For KP reporting, stock changes are reported in three HWP pools (sawn wood, panels and paper), but only those that are derived from domestic harvesting of forest lands are included. HWPs derived from harvesting Afforestation or Reforestation lands (AR lands) are tracked from 1990, while those from Forest Management lands (FM lands) are tracked from 2013 onwards. Products arising from deforestation are excluded as required in KP reporting. Above-ground biomass loss by activity and sub-category (e.g. harvest of AR lands) is obtained from the LUCAS forest model [13], so that HWP inflow can be attributed correctly for reporting and accounting. The LUCAS forest model combines information from field-based inventories and wall-to-wall satellite-based mapping and is used to support New Zealand’s international reporting requirements under the UNFCCC and the KP. Information on afforestation, deforestation and harvesting is used to simulate the development of forests over time and estimate stocks and stock changes in the forest carbon pools [15].

The KP spreadsheet model and UNFCCC HWP reporting model were combined so that New Zealand’s reporting under both the UNFCCC and KP now use the three Tier 1 HWP categories—sawn wood, wood-based panels, and paper and paperboard.

### NZ HWP model inputs

#### FAO Data—production, exports and imports

The NZ HWP Model uses the production and trade data provided by the NZ Ministry for Primary Industries to FAOStat from 1961 to the present time [16]. The use of first order decay in the model requires data from earlier than 1961 to initialise the HWP pool as at 1990 for UNFCCC reporting. The IPCC model uses a backfilling approach based on a variable, *U*, which links growth in production and trade to population growth rate. Initially the default *U variable* value for Oceania [3] was implemented in the NZ HWP model. However, New Zealand has been a source of wood products for export since European colonisation in the early 19^th^ Century, with production linked to demands from overseas as well as domestically. A large-scale, export-focussed wood processing industry was established in the mid-twentieeth Century [17]. Data on HWP production and trade was therefore obtained from historical records as an alternative and implemented as an option (see Additional files 1 and 2). This has no impact on KP reporting for New Zealand, because this begins with inflow from 1990 for post-1989 (AR) forests and 2013 for pre-1990 (FM) forests using the Production approach, ignoring the pre-existing pool of HWPs derived from earlier harvesting.

The New Zealand government commissions modelling of future planted forest management (including afforestation, deforestation, harvesting and replanting). This modelling provides projections of removals and emissions under low, medium and high emissions scenarios to inform policy development but excludes the HWP pool. There are no official estimates of future HWP production and trade at a product level. Assumptions about the future proportion of logs allocated to domestic processing, current and future export markets, and the broad product mix can be defined by the user in the NZ HWP Model if future projections of the HWP contribution are required. These future projections—particularly of individual products—are naturally indicative only and importantly are replaced by actual available evidence for the reporting under the UNFCCC.

#### Wood density

New Zealand’s production, consumption and exports of wood are dominated by a single species—radiata pine (*Pinus radiata* D. Don)—for which wood properties have been extensively researched. It is known that radiata pine wood density varies widely between stands, between trees and within trees, driven by factors such as temperature, soil fertility, genetic stock, silviculture and age [18]. An early attempt to quantify carbon stored in New Zealand forest products used a regression equation to produce whole tree wood density estimates at the end of a typical 28-year rotation of 0.39 to 0.40 oven dry tonnes (odt) m^−3^ [11]. A survey of wood properties suggested an average whole tree basic density of 0.38 odt m^−3^ (range 0.33 to 0.45) [19], while the 1995 NZIF Forestry Handbook lists an average value of 0.42 odt m^−3^ at merchantable ages [20]. In the absence of nationally-representative sampling of annual HWP production, the value of 0.42 odt m^−3^ has been used as a country-specific value for production and exports of coniferous sawnwood and veneer sheets, while values for production and exports of non-coniferous sawnwood and imports of all sawnwood and veneer sheets were estimated based on the mix of species. IPCC defaults were used for panel products (Table 2).

**Table 2:** Density and carbon fraction assumptions

	Oven dry density t m^−3^			Carbon fraction IPCC 2006 default
	IPCC 2006 default	NZ-specific (Production)	NZ-specific (Imports)	
Sawnwood (coniferous)	0.45	0.42	0.4	0.5
Sawnwood (non-coniferous)	0.56	0.5	0.7	0.5
Veneer sheets	0.505	0.42	0.7	0.5
Plywood	0.542	–	–	0.493
Particle board/OSB	0.596	–	–	0.451
Fibreboard, compressed	0.739	–	–	0.426
Insulating board/other fibreboard	0.159	–	–	0.474

#### Carbon fraction

The IPCC default carbon fractions were used for all products (Table 2). Maclaren and Wakelin used a value of 0.496 for all planted forest stand components in New Zealand [11]. Beets and Garrett found an average carbon fraction of 0.51 g C g^−1^ dry matter in needles and branches, 0.54 g C g^−1^ dm in bark and 0.50 g C g^−1^ dm in stem wood and roots for radiata pine in New Zealand [21]. These values are used for carbon stock estimates in plantation forests [22, 23].

#### Bark

The 2006-Guidelines provide a default bark expansion factor of 1.13, to estimate total harvest including bark from under-bark FAO data. This is an average of estimates for hardwoods (1.15) and softwoods (1.11). The softwood value was used, as this corresponds to the modelled radiata pine estimate for New Zealand [24].

#### Product category lifespans

Radiata pine is notable for its application in a wide range of end uses with very different half-lives within the same broad product categories. No detailed studies of the lifespans of products in New Zealand are available, but where estimates have been made, they broadly conform to the IPCC default half-life values (sawn timber 35 years; panels 25 years; paper and paper-board 2 years). Buchanan and Levine used an “average life” of 1 year for fuelwood and waste, 3 years for paper and 40 years for solid wood, based on the international literature [25], while Maclaren and Wakelin used the following in-use lifespans, based largely on experts’ judgements [11]:

- Long term (80-year lifespan): posts and poles.
- Medium term (50-year half-life): building, furniture and other manufacturing (assessed as 62% of panels and sawn timber).
- Short term (1-year half-life): pulp, paper, plus remaining 38% of panels and sawn wood used for concrete form-work, packaging etc.

An estimate of 90–110 years has also been derived for the lifespan of New Zealand houses [26].

IPCC default half-lives were applied to wood products produced in New Zealand whether used domestically or not, under the assumption that it would be less economically viable for New Zealand processors to produce and export shorter-lived, lower-value products. Traditionally Australia was the main market for sawn timber where it was used in a similar way to the domestic market. In the absence of further information this assumption was extended to all exported products, but based on new research it cannot be applied to solid wood products derived from exported logs. Manley and Evison examined the conversion and use of New Zealand-grown logs in the main markets of China, South Korea and India [27, 28]. Overall weighted half-lives for all New Zealand export logs in these countries was estimated as 6.6, 18 and 2.5 years respectively, reflecting differences in conversion rates, the use of processing residues, end-use applications and recycling. These estimates are available in the model as an alternative to the assumption that export logs are converted to products in the same proportions and with the same half-lives as those processed locally. Exported wood chips and pulp can be optionally included under the assumption that they are converted to paper, with the default IPCC half-life of 2 years applied in all cases.

#### KP activities—Afforestation/Reforestation, Forest Management and Deforestation

**Fig. 2:**
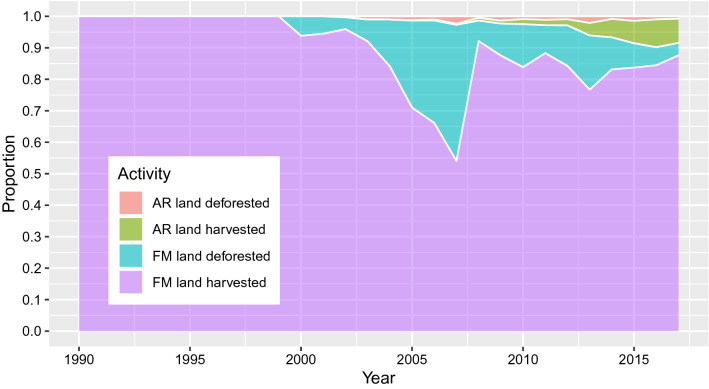
Proportion of annual production derived from deforestation and harvest activities in two forest sub-categories: Pre-1990 planted forests (FM land) and post-1989 planted forests (AR Land)

KP reporting requires that a distinction is made between HWPs derived from harvesting of Afforestation/Reforestation (AR) lands, Forest Management (FM) lands, and Deforestation lands. New Zealand’s HWP production statistics do not capture this information, but emissions from harvesting and deforestation by AR and FM separately are captured annually in the LUCAS forest model for greenhouse gas inventory reporting [13]. These estimates are based on activity data obtained for harvesting and deforestation in each forest sub-category, combined with per hectare carbon pool estimates derived from an unbiased national permanent sample plot network. This information derived from the forest model (Fig. 2) was used to set the annual proportions for HWP inflow in the HWP model, with the assumption that each activity produces the same mix of HWPs. Deforestation most commonly occurs at the end of a normal rotation of radiata pine, limiting the error introduced by ignoring product mix and wood density variation due to deforestation of other species or at other ages. HWPs derived from deforestation are excluded from accounting.

HWPs derived from non-forest sources must be excluded from KP reporting, but the proportion of New Zealand’s HWP production arising from these sources is likely to be insignificant so this has been ignored. In contrast, New Zealand’s short rotations and dynamic land use change in both directions between planted forests and agricultural land mean that HWPs derived from AR land and deforestation cannot be ignored.

#### Solid waste disposal sites (SWDS) reconciliation

The NZ Waste Sector model includes input of the amount of wood and paper deposited annually in managed landfills, unmanaged landfills, farm landfills and uncategorised landfills. The total should be equal to the amount of HWPs discarded domestically, less any HWPs recycled back into HWPs, less any discarded HWPs that are not sent to landfills. The 2006-Guidelines state that all CO_2_ released from HWP’s must be reported in the AFOLU sector. This means that Waste Sector estimates of CO_2_ emissions do not include CO_2_ lost from HWPs in landfills, although they do include CH_4_ emissions from this source. Instead, stock changes in HWPs in SWDS are calculated in the waste sector model but added to the HWP model to estimate two of the variables used for the three accounting approaches (variables 1B and 2B, Table 12.1 in IPCC [3]). These variables distinguish between all discarded wood and wood derived from domestic harvest only, for alignment with the system boundaries of the accounting approaches (see [6]). The 2006 Guidelines also suggest a reconciliation approach that uses estimated discards from the HWP pool after taking paper recycling into account and HWP inflow to SWDS to estimate the proportion of discarded HWPs that enter landfills. Variables used in the New Zealand waste sector model [13] are:

- DOC (Degradable Organic Carbon as % of wet material deposited): Paper 0.4; Wood 0.43.
- DOCf (Fraction of DOC dissimilated): 0.5.
- MCF (Methane Correction factor) 0.42 to 1 depending on landfill type.

### Sensitivity analysis

A sensitivity analysis of the NZ HWP model was conducted by comparing the results for the three UNFCCC approaches under variations of data and assumptions:

- Data back-filling method (U variable or country-specific data);
- Product conversion variables (IPCC default vs country-specific values);
- Contributions of imported wood to paper and paperboard.
- Inclusion or exclusion of HWPS made from exported roundwood, and assumptions made about exported roundwood conversion to HWPS and half lives;
- Inclusion or exclusion of paper made from exported chips and pulp;
- Inclusion or exclusion of posts and poles.

### Uncertainty assessment

Uncertainty was calculated for the contribution of HWPs under the Production approach by following the IPCC 2006-Guidelines approach based on propagation of uncertainties [3]. IPCC default uncertainty estimates were accepted for the FAOStat data and half-lives (Table 3). To reflect the greater certainty on the parameters to convert volumes to carbon weight that exists for New Zealand’s intensively managed, well-studied plantations that are dominated by a single species, the default uncertainty values were reduced (Table 3).

**Table 3:** Uncertainty estimates used in HWP pool calculations

Activity data and emissions factors	Uncertainty %	Source
HWP Production, import and export data	15	IPCC default ([3])
Product volume to weight factors	10	Country-specific (IPCC default 25%)
Oven dry product weight to carbon weight	5	Country-specific (IPCC default 10%)
Discard rate	50	IPCC default ([3] Table 12.6)

## Results

### HWP production

**Fig. 3:**
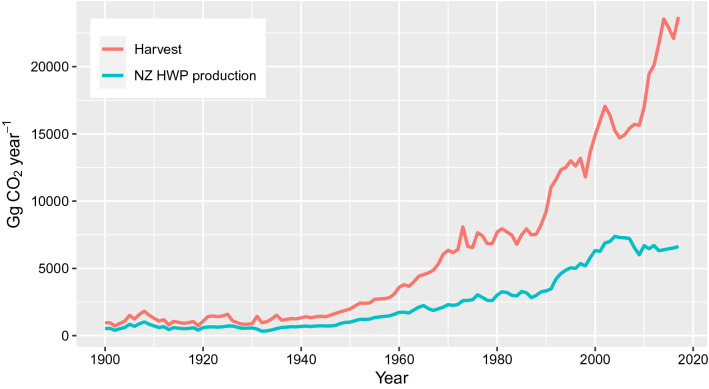
New Zealand annual domestic harvest and total domestic HWP production 1900–2017

**Fig. 4:**
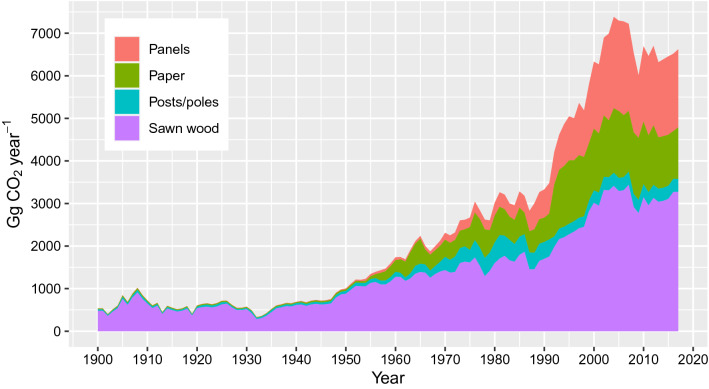
New Zealand domestic HWP production by semi-finished category

Harvesting and the domestic production of HWPs has increased in New Zealand since 1900 due to the development and expansion of a plantation forest industry (Figs. 3 and 4). Harvesting from natural forests has declined over this time and now makes up just 0.1% of the total roundwood removed [29]. The plantation harvest is mainly targeted at export markets with a small component aimed at the domestic market. Domestic production of HWPs has not kept pace with the increasing harvest, with the difference being mainly due to the fact that New Zealand is one of the world’s largest exporters of softwood logs. The gap between the annual harvest and domestic HWP production also reflects processing residues that are not incorporated into HWPs, including waste and fuel.

### HWP contribution to net emissions under three accounting approaches

**Fig. 5:**
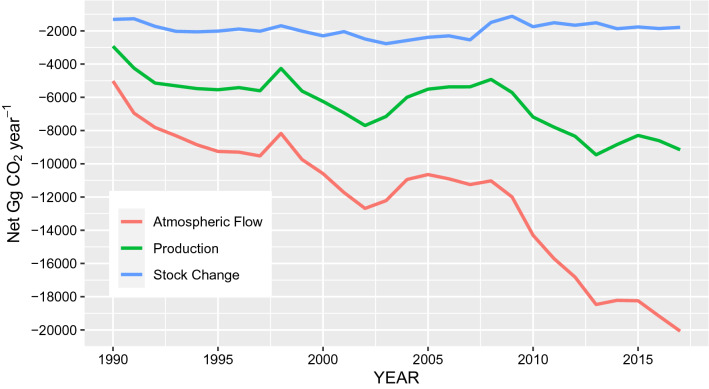
HWP Contribution to New Zealand’s net emissions by IPCC accounting approach

Figure 5 shows the wide divergence of the HWP contribution to net emissions under three accounting approaches. Under the Stock Change approach, the annual increase in the size of the HWP pool is estimated to be low and relatively stable between 1990 and 2020, as domestic demand for HWPs is similarly low and stable. This also means that emissions to the atmosphere within New Zealand are low as any emissions from exported HWPs are attributed to the importing country rather than accounted for within New Zealand. As a result, net removals attributed to New Zealand under the Atmospheric Flow approach are high. Because the proportion of the harvest destined for export markets increases through the time period, the gap between the Stock Change and Atmospheric Flow approaches widens, as it is advantageous for New Zealand to account for carbon uptake by forests within New Zealand without accounting for the emissions from exported products that occur offshore. This ignores the potential for wood exported from New Zealand to be re-imported as finished products. If more finished HWPs are imported than exported, then Tier 3 accounting for finished HWPs would increase the Stock Change contribution and decrease the contribution under Atmospheric Flow, bringing them closer together. Under the Production approach, New Zealand assumes responsibility for emissions from all wood grown domestically, whether consumed locally or not, while HWPs imported into New Zealand are accounted for by the country of origin, resulting in a HWP contribution midway between the other two approaches.

### Sensitivity analysis

i. Country specific activity data and product conversions

The Base scenario assumed data back-filling from 1900 using the default Oceania IPCC *U* variable and country-specific product conversions. Table 4 shows that using country-specific historic data in place of the *U* variable estimates (S1) has little impact under the Atmospheric Flow and Production Approaches, especially by 2017 when little pre-1960 material remains in-use. There is a greater effect under the stock change (SC) approach (3.1%) than for atmospheric flow (AF: 0.8%) and Production approach (P: 0.8%). The impact of the use of a variable wood density parameter also varied by approach but was generally low (S2). This Scenario 2 reflects the historical mix of species harvested and traded, with generally higher wood density than radiata pine. Using IPCC default product conversions (S3 and S4) resulted in a much greater HWP contribution across all approaches because the default coniferous wood density is higher than for radiata pine and IPCC default products assume a higher contribution from non-coniferous species with greater wood density.

**Table 4:** Percentage difference in HWP contribution relative to the Base scenario

Scenario	Backfill method 1900–1960	Product conversion	Year	SC (%)	AF (%)	P (%)
Base	Oceania *U*	NZ-specific	1990	–	–	–
S1	NZ data	NZ-specific	1990	3.1	0.8	0.9
S2	NZ data	NZ (variable D)	1990	0.3	0.8	− 0.4
S3	Oceania *U*	IPCC default	1990	1.8	3.6	3.3
S4	NZ data	IPCC default	1990	5.1	4.4	4.3
Base	Oceania *U*	NZ-specific	2017	–	–	–
S1	NZ data	NZ-specific	2017	1.3	0.1	0.2%
S2	NZ data	NZ (variable D)	2017	2.5	0.5	− 0.
S3	Oceania *U*	IPCC default	2017	4.2	6.4	6.3
S4	NZ data	IPCC default	2017	5.6	6.5	6.4
Base	Oceania *U*	NZ-specific	Change 1990–2017	–	–	–
S1	NZ data	NZ-specific	Change 1990–2017	5.0	0.2	0.3
S2	NZ data	NZ (variable D)	Change 1990–2017	9.5	0.6	− 0.2
S3	Oceania *U*	IPCC default	Change 1990–2017	15.7	8.5	9.2
S4	NZ data	IPCC default	Change 1990–2017	20.9	8.7	9.5

Stock change (SC), Atmospheric Flow (AF) and Production (P) Approaches under a range of scenarios varying in product conversions and backfilling data. Positive values increase the HWP pool contribution (i.e. reduce net emissions)

For reporting based on the KP Production approach and with a projected reference level, inherited emissions (and hence backfilled estimates) are irrelevant so the impact of these scenarios is limited to the effect of the use of IPCC product conversion factors rather than NZ-specific factors (S3 and S4). The use of default factors increases the sum of removals over the commitment periods (2008–2012, 2013–2020 and 2021–2030) by 6–7%.

ii. Domestic share proportion

The share of industrial roundwood that is produced domestically (f_IRW_ in IPCC 2006) is very high, averaging 99.96% from 1990 to 2017. The domestic share for pulp (f_PULP_) is slightly lower at 98.11%. These factors are multiplied together to give the proportion of paper and paperboard that is produced from domestically grown wood. In practice New Zealand’s imported roundwood is dominated by hardwood poles and railway sleepers that are unlikely to be further processed into HWPs or pulp, so f_IRW_ could be assumed to be 1 (100%). This has a negligible effect on the HWP contribution (< 0.1%).

iii. Exported roundwood

The Tier 1 UNFCCC reporting method assumes that roundwood exported by New Zealand is converted to products in the same proportions as for domestic processing and with the same half-lives. Logs imported from New Zealand form only part of total log supply in these markets and are not necessarily used in the same way as logs from other suppliers, because of their species, age and plantation origin. The use of an average country-specific half-life for each importing country would therefore be inappropriate. The Base scenario used in the NZ HWP Model instead assumes the end uses and half-lives reported by Manley and Evison [27, 28] for New Zealand’s largest log-importing markets. Assuming these half-lives rather than the IPCC defaults has no effect under the Stock Change or Atmospheric Flow Approaches because emissions from HWPs derived from exported logs are not included in these approaches. Under the Production Approach the higher conversion rates achieved by overseas processing compensates to some extent for life spans that are shorter than those for HWPs produced in New Zealand. The proportion of exported logs taken by each importing country changes over time in the model, but it was assumed that end uses have remained the same in each country. The use of export market-specific end-uses and half-lives resulted in a sink that was 16% lower in 1990 but 16% higher in 2017 than if domestic assumptions were used. Under KP reporting the sink was lower in 2008–2012 and 2013–2020 under domestic end use and half-life assumptions. If export logs are excluded from reporting altogether the sink is 54% and 61% lower for the two commitments periods respectively, reflecting the high proportion of the annual harvest that is exported in log form.

iv. Exported pulp and wood chips

The contribution to the HWP pool from paper and paperboard made from exported pulp and wood chips was not included in the Base scenario. If included in KP reporting they increase the HWP contribution by 6% in 2008–2012 and 4% in 2013–2020. This assumes a chip to paper conversion rate of 0.7 and pulp to paper of 0.96.

v. Posts and poles

Posts and poles are widely used in New Zealand for fencing, utility poles, retaining walls, house foundations and horticultural framing, and can have very long lifespans in some of these uses. According to IPCC 2006 they are excluded from accounting to avoid double-counting, as it is not clear whether material recorded in national statistics as “other industrial roundwood” will be further processed (e.g. into sawn timber or panels) or used directly as a finished product [3]. Posts and poles have therefore been excluded from the Base scenario, but in New Zealand’s case double-counting is unlikely so the sawn wood half live was applied as a scenario in the absence of specific information on the actual mean lifespan of posts and poles in use. Given annual production of posts and poles of 400,000 m^3^, the KP first and second commitment period sinks increase by 3% and 2% respectively.

### Uncertainty assessment

Estimated overall uncertainty for the net change in the HWP pool in 2017 is given in Table 5. The uncertainties for the total are product weighted and therefore reflect New Zealand’s unique HWP structure. The uncertainty associated with the stock change in the paper and paperboard pool is large in percentage terms because the change is relatively small. The stock change in paper and paperboard is only 2% of the total stock change in HWPs, so the high uncertainty does not have a major impact on overall uncertainty.

**Table 5:** Uncertainty assessment of net change to HWP pool in 2017

Product	Net change 2017 Gg C	% uncertainty
Sawn wood	2077	31
Panels	1273	34
Paper and paperboard	80	493
Total	3431	25

## Discussion

The results confirm that the different IPCC HWP accounting approaches give very different net emissions contributions for New Zealand—a country with a small domestic market relative to high levels of wood exports. Other net exporters of wood products have also reported the HWP pool as a top ten key category in terms of the level and trend of net emissions since 1990 including Sweden (based on the Production approach) [30] and Canada (based on the Simple Decay approach applied to harvest removals) [31].

The NZ HWP Model was adapted from the IPCC HWP model primarily to meet New Zealand’s international reporting requirements. In this it is similar to C_HWP [32] chosen as the exemplar of the IPCC approach in the review of HWP models by Jasinevičius et al. [9]. The review authors list ten modelling components found in HWP models, and indicate that those most frequently missing are:

1. decomposition in landfills;
2. recycling;
3. the existence of a value chain;
4. the product substitution effect.

With regard to point 1, the NZ HWP Model follows the IPCC approach with half-lives reflecting life spans of products in use, and with the pool of long term HWPs in SWDS taken into account. The model includes the suggested reconciliation with the IPCC SWDS model based on landfill deposition data. This indicates that the models are broadly compatible if the proportion of discarded wood products deposited into landfills is about 61%. While this is similar to the value reported by Zhang et al. for China (63% deposited in unmanaged deep fills, 37% burned) [33] only 10% of discards from the Australian pool of HWPs in-use are estimated to be landfilled, with 60% recycled instead [34]. Further work is required to reconcile New Zealand’s waste and HWP models.

The recycling example (point 2) given in Jasinevičius et al. [9] concerns the use of waste wood for panels or as fuel. The use of processing waste in panel products is already captured in the FAO data, but product cascade (e.g. conversion of discarded sawn timber to panels) is not. The NZ HWP Model does capture this for products made from exported logs when assigning a half-life. For example, the half-life assigned for temporary construction timber reflects its use as timber and also subsequent use as panels or paper for the proportion that is recycled [28]. This approach could be extended to products produced domestically, although information on recycling is not currently available.

The value chain component (point 3) identified by Jasinevičius et al. [9] was intended to indicate a material balance flow from the forest through to release of carbon back to the atmosphere. As indicated in Fig. 1, this is not a feature of the Tier 1 IPCC method and is not enforced by the NZ HWP Model for reporting. However, a material balance approach is applied to export logs, and is a more convenient way to carry out scenario analyses for future net emissions targets, given that it is easier to project future harvest and export log volumes than the production and trade of individual product sub-categories. A “value chain component” could also be interpreted to refer to the capture of greenhouse gas emissions along the value chain (e.g. from harvesting and transportation of products). These are reported within the Energy sector of the greenhouse gas inventory rather than attributed to land uses, and in the case of forestry in New Zealand are small compared with carbon uptake over a rotation [35].

Product substitution effects (point 4) are not included in the NZ HWP Model which is a more serious limitation for policy analysis, given that the substitution benefits of HWPs are estimated to be greater than the direct carbon storage benefits [36]. The use of models that do not include the impact of product substitution has led to incomplete analysis of the relative merits of production forests and unharvested conservation forests in the transition to a low net emissions economy in New Zealand [37].

A limitation of the NZ HWP Model is the lack of any corroboration of the size of the HWP pool estimated through the flux approach described here with an inventory approach. Research into the life spans of HWP sub-categories produced in New Zealand in both domestic and export markets (e.g. timber used for packaging) is also required to determine whether the proportions and half-lives by end use are similar to the global averages used to derive the IPCC default values. Product sub-categories could be explicitly modelled rather than used as the basis for creating weighted half-lives. This would make it easier to allow for change in parameters over time. Currently half-lives are fixed over time for key export markets, although the share of each market and hence the mean lifespan does change over time.

New Zealand’s Emission Trading Scheme (NZETS) is the main policy tool used by the New Zealand government to drive behaviour towards reducing net emissions and achieving domestic and international climate change targets. Through the NZETS, afforestation is incentivised by the potential to earn emission units through carbon uptake in forests established after 1989. Carbon storage in wood products is not currently recognised, with harvesting assumed to result in an instantaneous emission. Alternative policies to incentivise greater use of wood products in longer-lived applications (e.g. by rewarding forest growers, processors or consumers) are under investigation.

## Conclusions

The use of country-specific parameters in place of IPCC defaults results in a smaller but more realistic HWP pool sink for New Zealand. This is largely because the dominant planted forest species is a softwood of medium density and often applied in end-uses with relatively short lifespans, including paper and paper board, concrete formwork and packaging, particularly in export markets. New Zealand is a small economy with a significant forest product export industry, so end-uses in offshore markets have a major influence on changes in the HWP pool. End-uses and lifespans both in New Zealand and overseas will need to be regularly assessed in future to capture any changes, and further disaggregation of the semi-finished HWP end uses within New Zealand would be useful to improve accuracy.

The NZ HWP Model currently meets the needs for reporting under the UNFCCC and Kyoto Protocol. While it is also capable of providing projections to 2030 for reporting National Communications and Biennial reports under the UNFCCC, a material balance approach based on projections of harvest volume is more convenient for this purpose. More extensive analyses that includes the benefits of avoided emissions through product substitution and life cycle emissions from the forestry sector are an important future requirement to fully assess the potential contribution of forests and forest products to climate change mitigation and a low emissions future economy in New Zealand.

## Supplementary information

**Additional file 1.** Estimates of production and trade quantities from 1900 to 1960.

**Additional file 2.** Description of production and trade quantities from 1900 to 1960.

## Data Availability

Forest products production and trade data are available from FAOStat. Country-specific backfilling data are available in additional files.

## Abbreviations

AR: Afforestation and Reforestation (Kyoto Protocol Article 3.3 activity since 1990)
D: Deforestation
FAO: Food and Agriculture Organisation of the United Nations
FM: Forest Management (Kyoto Protocol Article 3.4 activity)
GHG: Greenhouse gases
HWP: Harvested wood products
IPCC: Intergovernmental Panel on Climate Change
KP: Kyoto Protocol
NZETS: New Zealand Emissions Trading Scheme
SWDS: Solid waste disposal sites
UNFCCC: United Nations Framework Convention on Climate Change
