# Supplementary material for: Estimating New Zealand’s harvested wood products carbon stocks and stock changes
Source: Carbon Balance Manag. 2020 May 21;15:10. doi: 10.1186/s13021-020-00144-5 (PMC7243304; doi:10.1186/s13021-020-00144-5)
Supplement: Supplementary file 2 — Additional file 2. Description of production and trade quantities from 1900 to 1960. [file 13021_2020_144_MOESM2_ESM.docx]

**Additional file -** **New Zealand-specific data for backfilling 1900-1960**

## Production 1900-1960

The default backfilling approach uses equation 12.6 from IPCC (2006):

$$V_{t}=V_{1961} .e^{[U . \left( t-1961 \right)]}$$

Where:

V_t_ = annual production, imports or exports for a solidwood or paper product for year *t*, Gg C yr^-1^

*t* = year

V_1961_ = annual production, imports or exports for a solidwood or paper product for the year 1961, Gg C yr^-1^

U = estimated continuous rate of change in industrial roundwood consumption for the region that includes the reporting county between 1900 and 1961; = 0.0231 yr^-1^ for Oceania.

Industrial roundwood production from New Zealand forests was not generally reported in the early 20^th^ century. Annual estimates are available from 1951 in MPI data^[[1]](#footnote-1)^, and earlier estimates are available from Rhodes and Novis (2002)^[[2]](#footnote-2)^ and at five yearly intervals from 1921 (Levack 1979)^[[3]](#footnote-3)^. The five-yearly interval estimates were scaled according to available sawn timber estimates. From 1900 to 1920, production of roundwood was set at twice the production of sawn timber.

Production of sawn timber was reported in the New Zealand Year Book for 1916 and annually from 1918. Data was converted from board feet using a factor of 0.00236 where necessary. A range of year-end conventions were used in the past and no attempt was made to convert data to calendar years – the estimates should be seen as indicative. A breakdown by the main species (kauri, kahikatea, rimu, matai, totara, beech, radiata and others)^[[4]](#footnote-4)^ was also published and this was retained to allow a weighted-average wood density to be calculated. These Year Book estimates were used until 1951 when sawn timber production by species was available from MPI data.

For the period from 1900 to 1915, data was based on estimates of kauri sawn timber production in 1900 and 1904 provided by Thode (1983)^[[5]](#footnote-5)^, the relative production of different species from 1916-1920, and the knowledge that kauri (and total) production peaked around 1907. These estimates are indicative only, but certainly better that those produced using the default U variable approach. They have little impact on UNFCCC reporting due to the decay rate assumed, and no impact on Kyoto Protocol reporting.

Panel production is available from 1946 for plywood and fibreboard. Plywood production before 1946 was set at 10% of sawn wood production, which was the average for the 1946-1951 period. Peeler log production by species was available from 1971 but the weighted average density was similar to that calculated for sawn timber so the value derived for sawn timber was used. Particleboard was invented in Germany during the Second World War and was set to zero until 1959 when MPI data provided small volumes. A 1948 FAO commodity report on fibreboard stated that "*New Zealand has but one plant which up to this time produced only insulating board. Equipment for the manufacturing of hardboard has now been installed and is expected to be in operation in 1948*." Capacity was expected to increase from 11,000 to 20,000 tonnes (FAO 1948)^[[6]](#footnote-6)^. The production of insulating board was set at zero before 1920 then increased linearly to the FAOSTAT data point in 1961. MDF was not produced until 1976.

The first paper mills in New Zealand were in operation by 1900 but production was largely based on rags, old rope and other fibres as well as imported pulp. Wood-based pulp became more important in the 1940s with the development of the Whakatane board mill and much more so in the 1950s with construction of pulp mills at Kinleith and Kawerau. Given the short half-life assumed for paper, the production of paper and paperboard was simply set at 2% of sawn timber production from 1900 until 1940 when the MPI time series began.

Comparisons of backfilling based on New Zealand estimates and the default approach using the U variable are shown in Figure 1-1 to 1-3.


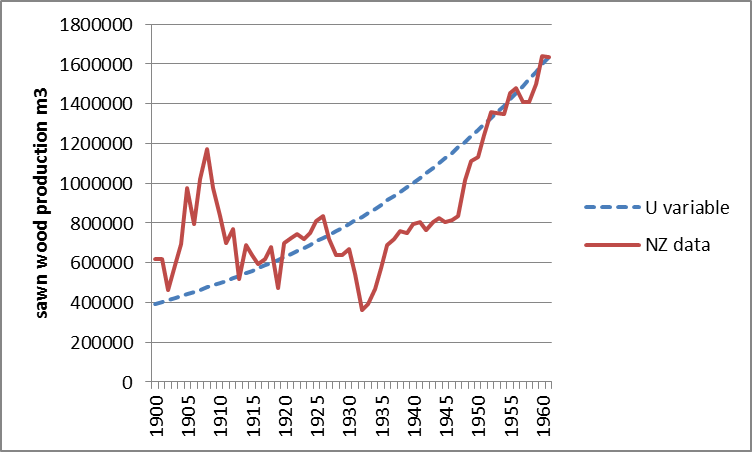


**Figure S1-1 Backfilling sawn wood production data**


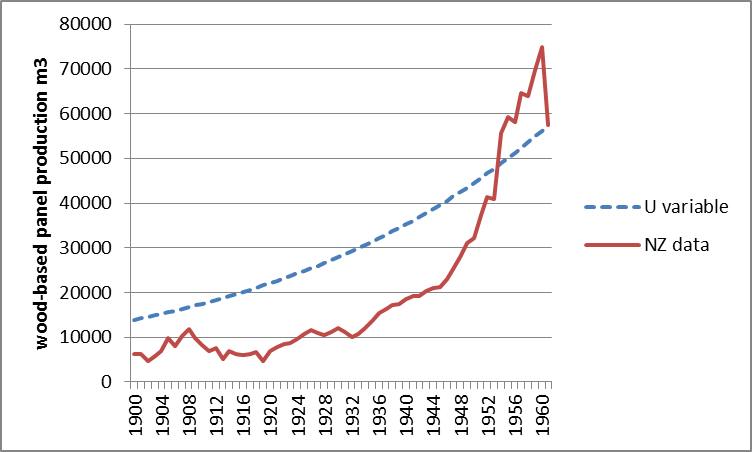


**Figure S1-2. Backfilling wood panel production data**


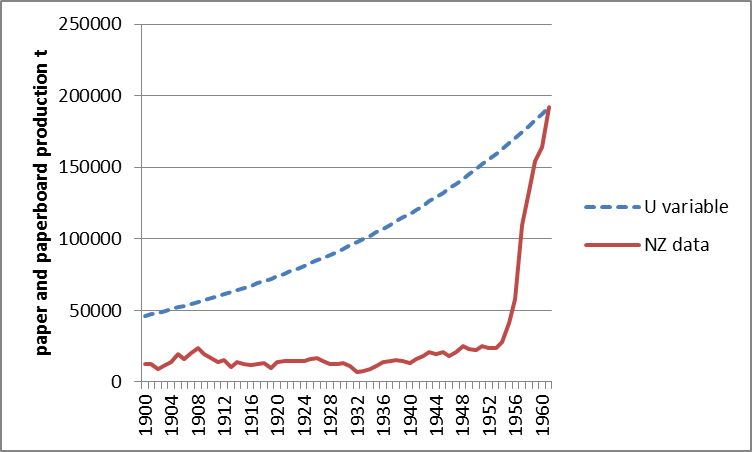


**Figure S1-3 Backfilling paper and paperboard production data**

## Imports 1900-1960

Imports data is available in the New Zealand Year Books^[[7]](#footnote-7)^ from 1900 but not always in a useful format. Imports in 1900 are given in Table 1-1 as an example – note in particular that logs, palings, posts and rails are all given as number of items, rather than a measured quantity. The list also includes finished HWPs.

**Table S1-1 HWP imports in 1900**

| Paper— Bags, coarse .. .. | 16 cwt. |
| --- | --- |
| Bags, other kinds .. | 2,275 cwt. |
| Butter-paper .. .. | 2,587 cwt. |
| Paperhangings .. | 985,496 pieces |
| Printing .. .. | 120,609 cwt. |
| Wrapping .. .. | 3,841 cwt. |
| Writing .. .. | 9,700 cwt. |
| Unenumerated .. | 1,628 cwt. |
| Laths and shingles .. | 682,000 sup. ft. |
| Logs .. .. .. | 1,836 No. |
| Logs, hewn .. .. | 3,461,106 sup. ft. |
| Palings .. .. | 472,741 No. |
| Posts .. .. .. | 12,628 No. |
| Rails .. .. .. | 1,350 No. |
| Sawn, undressed .. | 7,245,208 sup. ft. |
| Sawn, dressed .. .. | 139,720 sup. ft. |
| Unenumerated .. | .. |

Sawn timber data was obtained annually from 1924, with some estimates obtained for individual years before that. It was not always possible to distinguish between logs, poles, sleepers and sawn timber. MPI data on sawn timber imports by species from 1971 showed an even split between hardwoods and softwoods. The main softwoods were Douglas fir, western red cedar and redwood, with an estimated average weighted density of about 400 kg/m^3^. The main hardwoods were Eucalyptus, oak and tropical hardwoods, with an estimated weighted average density of about 700 kg/m^3^. A constant weighted density of 550 kg/m^3^ was assumed. These same species were also prominent in the pre-1971 data (Figure 1.4).

**
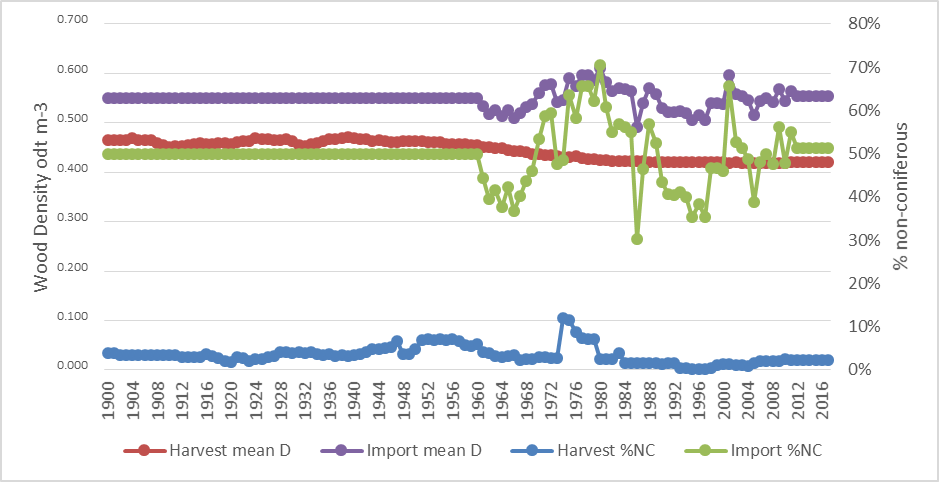
**

**Figure S1-4 Weighted mean density and percentage coniferous**

No information was obtained on fibreboard imports before 1955 or other panels before 1961, so linear interpolation was used to fill the time series, assuming 0 in 1920 for fibreboard and in 1900 for veneer and plywood. Approximate values for paper and paperboard were estimated for 1900 and 1904, with linear interpolation used to fill the time series until annual data was available from 1950. Pulp imports were assumed to increase linearly from zero in 1900 to the first data point available in 1955.

## Exports 1900-1960

Exports of timber began in the early days of European settlement at the turn of the 19^th^ Century. Kauri forests were heavily exploited in the 19^th^ century, with supply outstripping demand. Following a period of recession in the 1880s and 1890s production increased again, reaching a peak in 1907. As availability declined, attention turned to other species – principally kahikatea until supplies ran out in the 1940s then rimu. In 1931 radiata pine made up 2% of exported timber and this increased steadily to reach 50% in the 1940s. By the mid-1950s radiata made up over 90% of exported timber.

The main sawn timber market has always been Australia, taking as much as 99% of exports. The Pacific Islands and the United Kingdom were the other main markets for much of the 20^th^ Century. Exports of panels and paper began later, with the development of major pulp and paper mills in the 1950s to utilise the planted forest resource, and the development of MDF production in the 1980s.

Export data for sawn timber were available annually in the New Zealand Year Books from 1900. Some information on the proportion by species was also available, but for simplicity it was assumed that exports were the same mix of species as production. Australia was by far the main export market for sawn timber before 1961, and it has remained so.

Exports of other products did not begin until much later. Exports of pulp and paper and paper began in 1954 with paper (mainly newsprint) exports increasing rapidly. The Japanese export log market began in 1958 while the Japanese wood chip export market began in 1970.

A comparison of estimates of export data for 1900-1960 based on the IPCC U variable and use of New Zealand data is given in Figures 1-4 to 1-6.


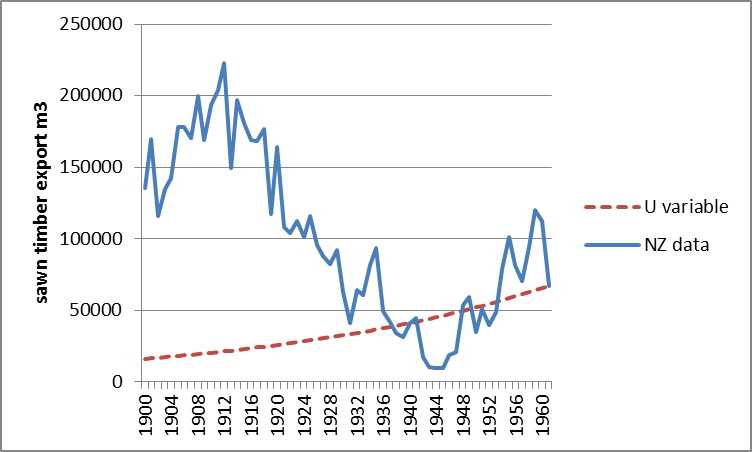


**Figure S1-4 Backfilling sawn wood export data**


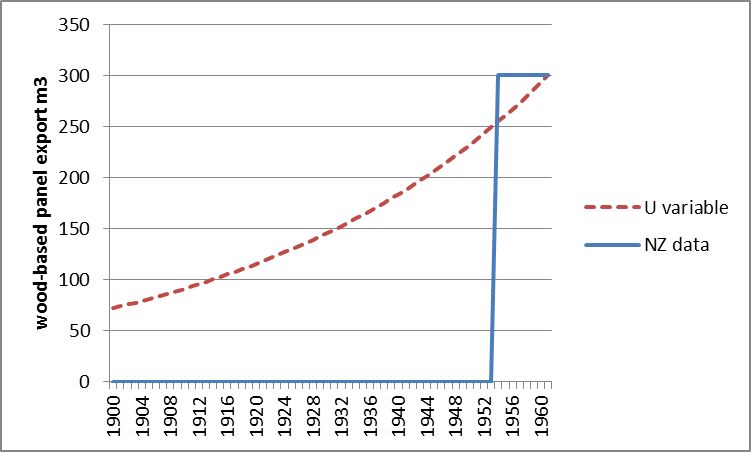


**Figure S1-5 Backfilling wood panel export data**


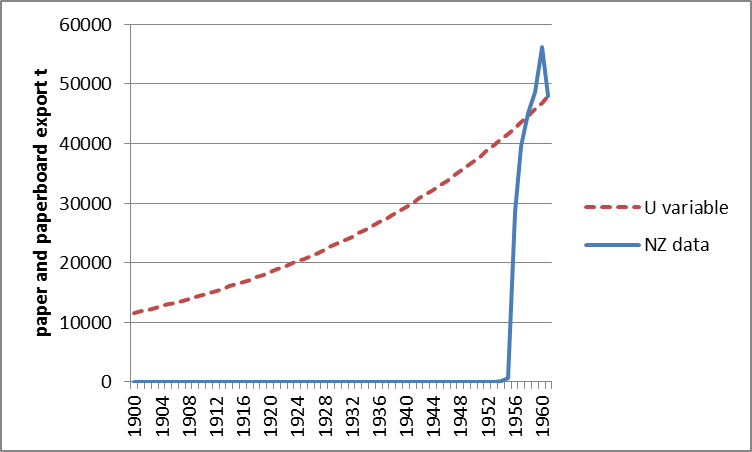


**Figure S1-6 Backfilling paper and paperboard export data**

Data backfilling is only required for UNFCCC reporting. Clearly the default *U* variable approach does not provide a very good estimate of New Zealand’s actual HWP production and trade, even allowing for the broad assumptions made in compiling the New Zealand data set. However, in practice the differences are minimised because by 1990 the assumed decay rate has greatly reduced the contribution from inherited emissions.

1. <https://www.teururakau.govt.nz/news-and-resources/open-data-and-forecasting/forestry/wood-processing/> [↑](#footnote-ref-1)
2. Rhodes, D., Novis, J., 2002. The Impact of Incentives on the Development of Plantation Forest Resources in New Zealand. MAF Information Paper vol. 45. Ministry of Agriculture and Forestry, Wellington. 61 pp. [↑](#footnote-ref-2)
3. Levack, H. H. 1979. Future national wood supply. New Zealand Journal of Forestry, 24, 159-171 [↑](#footnote-ref-3)
4. In many years the “other species” category was further broken down in up to 20 individual species. [↑](#footnote-ref-4)
5. Thode, P.J. 1983 Northland's forest history and present resources. New Zealand Journal of Forestry 28(2) 203-224 [↑](#footnote-ref-5)
6. FAO 1948. Fibreboard. Unasylva 2(4). [↑](#footnote-ref-6)
7. <http://archive.stats.govt.nz/browse_for_stats/snapshots-of-nz/digital-yearbook-collection.aspx> [↑](#footnote-ref-7)
